# Gut Susceptibility to Viral Invasion: Contributing Roles of Diet, Microbiota and Enteric Nervous System to Mucosal Barrier Preservation

**DOI:** 10.3390/ijms22094734

**Published:** 2021-04-29

**Authors:** Marcela Julio-Pieper, Alejandra López-Aguilera, Johana Eyzaguirre-Velásquez, Loreto Olavarría-Ramírez, Claudia Ibacache-Quiroga, Javier A. Bravo, Gonzalo Cruz

**Affiliations:** 1 Grupo de NeuroGastroBioquímica, Instituto de Química, Facultad de Ciencias, Pontificia Universidad Católica de Valparaíso, Valparaíso 2340000, Chile; a.lopezaguilera31@gmail.com (A.L.-A.); johana.eyzaguirre@gmail.com (J.E.-V.); javier.bravo@pucv.cl (J.A.B.); 2 APC Microbiome Ireland, University College Cork, T12 K8AF Cork, Ireland; lfernandaor@gmail.com; 3 Centro de Micro-Bioinnovación (CMBi), Escuela de Nutrición y Dietética, Facultad de Farmacia, Universidad de Valparaíso, Valparaíso 2340000, Chile; claudia.ibacache@uv.cl; 4 Centro de Neurobiología y Fisiopatología Integrativa (CENFI), Instituto de Fisiología, Facultad de Ciencias, Universidad de Valparaíso, Valparaíso 2340000, Chile; gonzalo.cruz@uv.cl

**Keywords:** gut, permeability, barrier, epithelium, submucosal neurons, gastrointestinal disorders

## Abstract

The gastrointestinal lumen is a rich source of eukaryotic and prokaryotic viruses which, together with bacteria, fungi and other microorganisms comprise the gut microbiota. Pathogenic viruses inhabiting this niche have the potential to induce local as well as systemic complications; among them, the viral ability to disrupt the mucosal barrier is one mechanism associated with the promotion of diarrhea and tissue invasion. This review gathers recent evidence showing the contributing effects of diet, gut microbiota and the enteric nervous system to either support or impair the mucosal barrier in the context of viral attack.

## 1. Introduction

There is an ever-increasing amount of evidence indicating that changes in the gut lumen affect the host on many physiological levels. In this regard, studies on gut microbiota, and their effects on local cell systems, such as the intestinal epithelium, gut-associated immune cells and enteric nerve cells, have gained a lot of momentum as a means to provide evidence concerning a wide array of diseases, including novel targets for treatment. Moreover, gut microbiota composition and behavior are susceptible to changes induced by diet, which can therefore contribute to health and/or disease. In the present review, we decided to look at another inhabitant of the gut lumen: viruses.

One remarkable feature of the human gut virome is its individuality and permanence over time. The stability of its composition is associated with a few consortia of viral genomes that are highly prevalent, persistent, and individual-specific [1]. Whereas these stable consortia represent a relatively small proportion of the whole gut virome, their ability to influence the rest of the local microbiota, and therefore the host’s health status, is certainly worthy of investigation.

## 2. What Types of Viruses Are Found in the Gut?

The human gut virome consists mainly of (1) bacteriophages that infect bacteria and archaea and (2) eukaryotic viruses with the ability to replicate in human cells. Small amounts of plant- and animal-derived viruses that are ingested with food can also be found in the gut contents [2]. The main families and genera of eukaryotic viruses found in the gut are summarized in Table 1.

Metagenomic tools have been valuable in characterizing the great majority of viruses that are not cultivable. It is now known that the eukaryotic gut virome expands progressively with age [3]. Contrary to what happens with gut bacterial microbiome, which can be highly coincident between family members, there is remarkable interpersonal variation of gut viruses, even between twins [4]. In addition, mother-to-infant transmission of the gut virome is minor when compared to that of gut bacterial microbiome [5]. Intrapersonal variation of viromes over time, on the other hand, is minimal [1,4]. In addition, the virome as a whole is able to influence the transcriptional status of non-infected cells (due to release of interferon and other cytokines by infected ones) without constituting viral disease [6], suggesting that some eukaryotic viruses could be considered residents of the human gut [4].

**Table 1:** Eukaryotic viruses of the gastrointestinal microbiota. Adapted from [7,8,9,10,11,12,13,14,15,16,17,18,19,20,21,22]. ^(a)^ This type of virus is also detected in healthy/asymptomatic hosts. ^(b)^ The etiologic role of this virus in human gastroenteritis remains uncertain.

Family	Genus	Clinical Manifestation	Reference
*Adenoviridae*	*Mastadenovirus* C, F and others	Gastroenteritis	[7]
*Anelloviridae*	*Anellovirus*	Enteritis ^a^	[8]
*Astroviridae*	*Astrovirus*	Gastroenteritis, meningitis, encephalitis	[9]
*Caliciviridae*	*Norovirus*	Gastroenteritis ^a^	[10]
	*Sapovirus*	Gastroenteritis ^a^	[11]
*Circoviridae*	*Circovirus*	Unclear	[12]
	*Cyclovirus*	Unclear	[12]
*Coronaviridae*	*Torovirus*	Gastroenteritis ^a,b^	[13]
*Parvoviridae*	*Bocavirus*	Gastroenteritis ^b^	[14]
	*Bufavirus*	Unclear	[15]
*Picobirnaviridae*	*Picobirnavirus*	Gastroenteritis ^a^	[16]
*Picornaviridae*	Enteroviruses including *Poliovirus* and *Echovirus*	Enteritis, neurologic syndrome	[17]
	*Parechovirus*	Gastroenteritis, respiratory infection, sepsis-like illness, CNS infection	[18]
	*Cardiovirus*	Gastroenteritis, respiratory infection, myocarditis	[19]
	*Salivirus*	Gastroenteritis	[20]
*Polyomaviridae*	*Polyomavirus*	Unclear	[21]
*Reoviridae*	*Rotavirus*	Gastroenteritis	[22]

## 3. The Enteric Virus Road

Pathogenic or not, in order to thrive in the gastrointestinal tract, a virus must endure several challenges that begin long before finding its cellular target (see [23] for a comprehensive review). The interhost phase, when a viral particle is exposed to variable and potentially aggressive environments, is the first of such challenges. Under optimal conditions of temperature, moisture and pH, human norovirus and poliovirus, for example, may persist for months; however, when exposed to more stringent environments, their stability can drastically drop to a few days [23,24,25].

**Figure 1:**
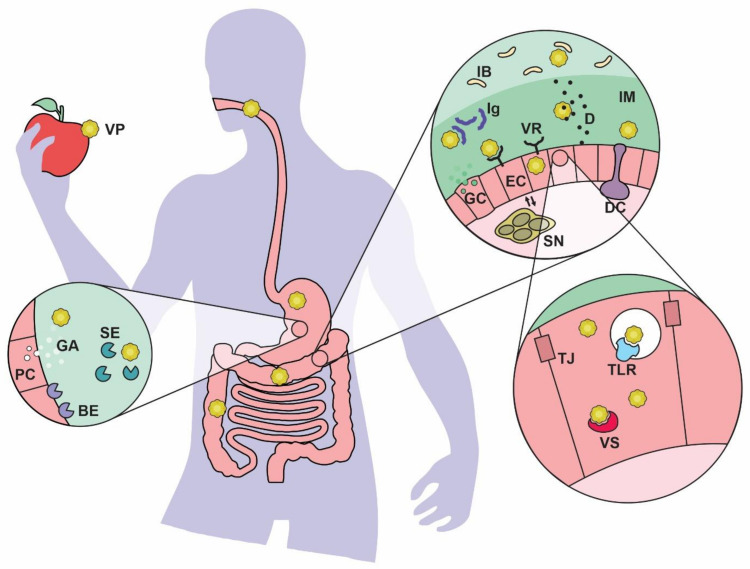
Barriers presented by the host to clear viruses travelling the gastrointestinal tract. Foodborne viruses can encounter diverse obstacles on their way to a target cell. The mouth, stomach and intestine contain physical, chemical and biological barriers that decrease viral count in the lumen and reduce accessibility to its target. In addition, intracellular antiviral mechanisms are designed to destabilize the virus and neutralize its ability to replicate. BE: brush border enzymes, D: defensins, DC: dendritic cell, EC: enterocyte, GA: gastric acid, GC: goblet cell, IB: intestinal bacteria, Ig: immunoglobulin, IM: intestinal mucus, PC: parietal cell, SE: soluble enzymes, SN: submucosal neurons, TJ: tight junction, TLR: toll-like receptor, VP: viral particle, VR: viral receptor, VS: viral sensor.

Upon oral ingestion of a virus, there are initial clearance mechanisms that are not specific to the host. Saliva and enzymes in the mouth, as well as mucus and cilia in the esophagus are designed to clear and/or inactivate microbial threats. As shown in Figure 1, the stomach’s low pH, additional digestive enzymes and bile salts are other barriers that viruses encounter as they navigate the gastrointestinal tract [24]. These chemical weapons do not affect all viruses the same; while enveloped viruses are known to be inactivated by bile salts, nonenveloped viruses can resist them [23]. Gastric and intestinal proteases, on the other hand, can increase the infectivity of some viruses, as has been shown for the Reoviridae family [26]. 

Viruses that have survived the above barriers must still penetrate a mucus layer that covers the luminal epithelium of the GI tract. In the stomach and colon, this gel-like substance is stratified into an external, less dense layer and an internal layer that is firmly attached to the epithelium and is more resistant to bacteria colonization, whereas in the small intestine, only one layer of loosely attached mucus has been described [27]. Mucus is formed by highly glycosylated proteins known as mucins, which, upon release from goblet cells (see Figure 1), are able to form a crosslinked network with high water-binding capability, giving the mucus its characteristic rheological properties [27]. Intestinal mucus contains a set of host-derived antimicrobial peptides known as defensins. Defensins’ ability to neutralize mucus viruses depends on several mechanisms, including extracellular aggregation, envelope disruption and receptor blocking [28]. Mucus also contains dimeric IgA and lactoferrin, which have antiviral properties reported in vitro and in animal models [23,29].

The gastrointestinal epithelium is lined by cells which are joined by tight junctions in order to prevent nonspecific passage of luminal material through the paracellular route. Various cell types can be found in this first cell layer; for a virus to enter cells of epithelial nature, namely enterocytes and goblet cells (see Figure 1), it must bind a specific viral receptor and undergo receptor-mediated endocytosis [30]. Consequently, cells expressing such receptors become potential virus targets. However, the gut epithelium also contains cells of immune relevance, such as dendritic cells, and M cells that can be found in specialized epithelium such as the mucosal lymphoid tissue. The latter are able to sample and transport luminal antigens [31]; therefore, viral uptake and transcytosis through M cells may occur without the need of viral receptor expression. Recently, it was reported that some viruses may facilitate tight junction dissociation, in order to bind additional co-receptor molecules that are located within the junctional complex [32]. Finally, opportunistic viral entry through gut epithelium that has been disrupted by a lesion or where tight junctions are destabilized, has been suggested as an additional mechanism of infection [33,34]. 

Once a virus gains access to the intracellular compartment, expression and replication stages can take place. In the case of RNA viruses, which are the majority among those targeting the gut, RNA is released from its coating. (+)RNA viruses are translated by host ribosomes, whereas (−)RNA viruses must first be transcribed into a positive RNA strand, which can then be translated. The newly translated polypeptide is then cleaved by viral proteases, generating a set of proteins required for viral genome replication and capsid organization. Finally, RNA copies are packaged and released, in a process that is accompanied by the death of the host cell [30]. Recently, some enterovirus species have been shown to also undergo non-lytic release [35]. It must be noted that upon contact with the host cell, the virus encounters a new variety of threats. Among them, viral sensors, which are surface and intracellular molecules from the host’s innate immune system that lead to the assembly of antiviral effector responses. They recognize molecular patterns either from the incoming virus or produced during its replication. In the gut, the sensors RIG-I, MDA-5 and NLRP6 activate type III interferons upon virus detection, which stimulate responses that act locally on the epithelial layer. TLR3, on the other hand, indirectly activates type I interferon (IFN) responses, which are active in the lamina propria [36]. IFNs can be released from infected cells and signal to neighboring cells, inducing an antiviral state that decreases their susceptibility to infection. In turn, viral proteases have evolved to cleave some pattern recognition receptors, dampening IFN-mediated responses [30]. Finally, epithelial cells are characterized by fast renewal which may be especially challenging for viruses heading to a secondary host tissue. Moreover, viral infection can result in accelerated epithelial cell turnover by a type I IFN-dependent mechanism [37], a response that may limit microbe spreading in the gut but also potentially affect tissue functionality.

## 4. General Effects of Enteric Viruses in Gut Health

Viral infection represents 75% to 90% of childhood acute infectious gastroenteritis in industrialized nations [38]. In adults, viruses are also the leading cause of foodborne diarrheal diseases [39]. Although other tissues (e.g., brain, lungs) may also be affected, as shown in Table 1, pathogenic gut viruses commonly cause gastroenteritis, with symptoms including vomiting and diarrhea. Infectious diarrhea develops as a result of one or more of the following mechanisms: hypermotility, enhanced epithelial secretion, decreased osmolyte absorption or increased epithelial permeability [24]. 

While there is plenty of evidence indicating the pathogenic effect of intestinal viruses, researchers are starting to focus on the potentially beneficial effects of some viruses on overall health. It has been reported that murine norovirus infection is able to revert some of the architecture loss and immunological disturbances that are observed in germ free mice or when conventional mice are subjected to antibiotic treatment [40]. Furthermore, it has been suggested that resident viruses can favor intestinal health by downregulating inflammation signals. This conclusion was drawn after observing that mice treated with a cocktail of antiviral drugs display higher pathologic scores to chemically-induced colitis, in comparison to mice that did not receive antivirals [41]. Moreover, upon activation of TLR3 and 7 (which recognize double-stranded and single-stranded viral RNA, respectively), dendritic cells release IFN beta, an anti-inflammatory cytokine [41]. 

**Figure 2:**
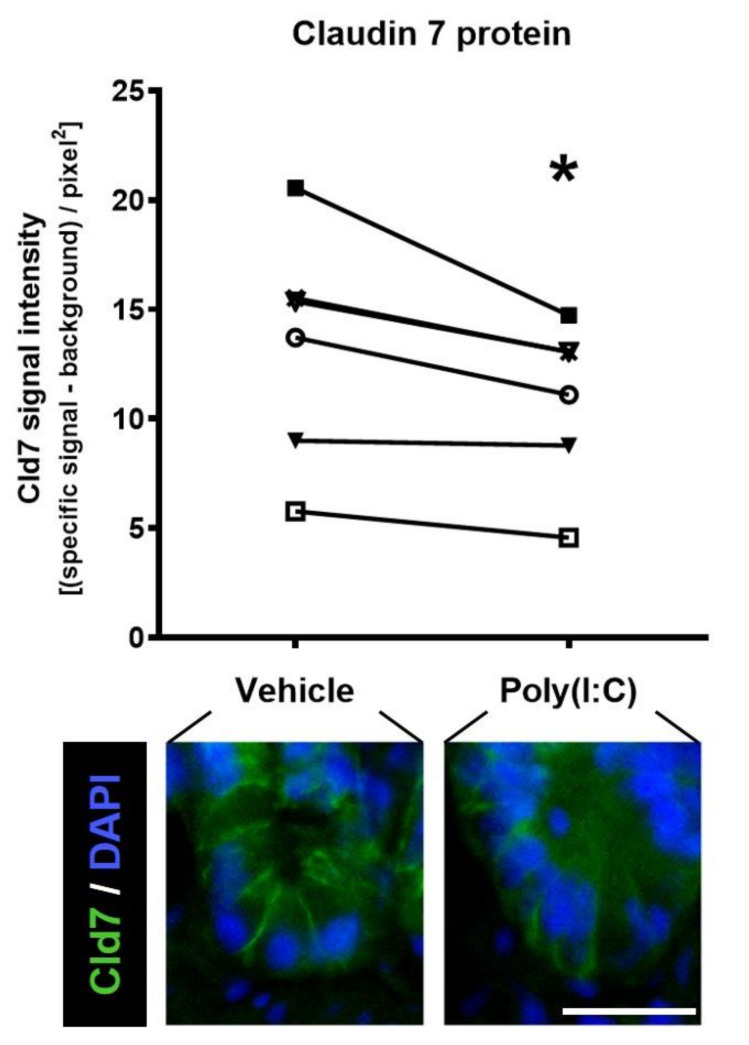
Claudin 7 protein in the ileal mucosa upon treatment with a TLR3 agonist. Rat ilea were treated ex vivo for 2 h with either vehicle (saline) or the TLR3 agonist, Poly(I:C) (200 μg/mL). Claudin 7 (Cld7), a component of tight junctions but also relevant for cell adhesion, was analyzed by immunofluorescence. In the graph, each line represents one rat (total *n* = 6) and connects the value obtained in the vehicle-treated portion of ileum with the value obtained in the corresponding Poly(I:C)-treated tissue. There was a loss of specific (lateral) signal in every case. Bar = 25 μm, * *p* < 0.05 by paired *t* test. Original data from Loreto Olavarría-Ramírez.

Viral molecules signaling through TLR may potentially protect the gut barrier by decreasing epithelial permeability. However, the notion of a microbe that is either completely pathogenic or always beneficial is probably outdated. For example, we have shown that Poly(I:C), a synthetic ligand that mimics viral double stranded RNA, reduces colon permeability to macromolecules when administered to rats intrarectally [42]. However, the same virus-like molecule increased ileal permeability when applied ex vivo (see Reference [42] and Figure 2 for previously unpublished data). Decreasing inflammation and diarrhea may be considered an evolutionary trait to extend the stay of a given parasite on the host’s intestinal tract. On the other hand, enhancement of epithelial permeability may potentially contribute to induce stronger immunity against the pathogen, a response that is highly sought after by vaccine developers [43].

## 5. Mucosal Barrier Changes under Viral Infection

A dynamic entity such as the mucosal barrier, readily adapts some of its features in response to the environment and viruses have taken advantage of this plasticity in diverse ways. While the ability of rotavirus to suppress IFN-mediated responses facilitates its infective process without inducing substantial inflammation of the bowel mucosa [44], noroviruses have deleterious effects on the intestinal epithelium, disturbing both cell function (i.e., increased turnover and enhanced apoptosis of enterocytes), as well as tissue architecture and physiology which results in reduced absorptive surface and increased permeability. The later was associated with reduced tight junction protein expression [45]. Also, increased numbers of intraepithelial lymphocytes were observed upon duodenal norovirus infection [46]. Murine astrovirus, on the other hand, is able to selectively infect the mucus-secreting goblet cells and functionally modify mucus in a way that suggests an alteration in host susceptibility to infection [47].

Some viruses (e.g., poliovirus) induce alterations of protein traffic and impact secretory pathways. This can result in a reduced expression of MHC class I molecules, as shown in vitro using a chimpanzee lymphoblastoid cell line [48]. In addition, a non-structural protein found in picornaviruses which, like viroporins, can induce the formation of pores in the host’s cell membrane, disrupt ion homeostasis [49], thus potentially interfering with vesicle-dependent secretory function. 

The gut mucosal barrier function is strongly influenced by environmental stressors including dietary changes and dysbiosis. Similarly, alterations of the enteric nervous system, a local regulator of intestinal physiology, can resonate on the gut barrier function. We will discuss the impact of viral infection on these three elements, but also how they may influence the mucosal susceptibility to viral attack. 

## 6. Interactions between Gut Bacteria and Potentially Pathogenic Viruses

Healthy gut microbiota has been generally syndicated as protective against pathogens. Bacteria are the most studied component of gut microbiota and their contribution to the host’s defense against pathogen invasion has been extensively shown. The mechanisms involved in protection against other bacteria, a phenomenon known as colonization resistance, can be indirect, such as displacement of noxious microbes due to competition for space and nutrients and/or enhancement of mucosal immune responses [50]. In addition, some commensal (as well as probiotic) bacteria have the ability to directly kill pathogenic entities [50]. 

Remarkably, a few viruses, including certain poliovirus and norovirus, require the presence of bacteria in order to become infective or to enhance their infectivity; others can be further benefited by these prokaryotic organisms (see [51,52,53] for a few comprehensive reviews). Bacterial surface products, such as lipopolysaccharides and other glycans, can act as stabilizers for viral particles, increasing their thermostability and resistance to disinfectant solutions containing bleach. Lipopolysaccharides also enhance poliovirus ability to attach to their eukaryotic cell targets [51]. In vitro studies suggest that viral genetic recombination can be facilitated by commensal bacteria acting as a reservoir for genetic material derived from multiple viruses [53]. Clinical evidence that point to (but do not discriminate between) the above mechanisms include the following: (1) the gut bacterial composition of patients affected by norovirus-related gastroenteritis was different from the one reported for asymptomatic norovirus carriers [54]; (2) a group of elderly residents from a health care facility who ingested a probiotic drink daily during at least two months displayed significantly less persistent fever upon winter season infection with norovirus, compared to elderly patients who did not ingest the probiotic bacteria [55]. The probiotic drink, containing *Lactobacillus casei* strain Shirota, was later shown to decrease the risk of infection, as well as the cell numbers of noxious bacteria including *Clostridium difficile* in elderly patients [56]; (3) children affected by gastroenteritis associated with viral-bacterial mixed infections had a significantly higher disease severity score than those children experiencing virus-only infection [57]. These reports support the notion that bacterial context has a clinically relevant impact on virus pathogenicity. This observation may also be true for other organs that are susceptible to viral infection, such as the liver [58]. 

Studies addressing the effects of viruses on commensal microbiota are still scarce. Gut dysbiosis has been reported in patients infected with HIV, HCV and HBV, although the main target of these pathogens is not the intestine [53]. Regarding gastrointestinal viruses, a study found a small cohort of norovirus-infected patients that displayed gut dysbiosis, consisting of significant loss of Bacteroidetes with an increase of Proteobacteria [59]. In the same investigation, it was found that a single operational taxonomic unit of *Escherichia coli* partially contributed to the increase in Proteobacteria [59]. A later study demonstrated the ability of human norovirus to efficiently bind the cell membrane and bacterial pili of certain species from the Proteobacteria phylum [60]. In a different context, the interaction of influenza virus and bacteria (i.e., *Streptococcus pneumoniae*) enhances pathogen in vivo fitness and translocation to the mouse middle ear, resulting in higher tissue bacterial burden and higher mortality than the observed without having pre-mixed the bacteria and the virus [61]. Whether commensal, non-pathogenic gut bacteria acquire infective features upon interaction with viruses remains to be investigated.

## 7. Malnutrition and Gut Viral Infections

The notion of an interaction between undernutrition and infection, initially proposed by Scrimshaw et al. in 1959 and later published as a World Health Organization monograph in 1968 [62], remained relatively unchanged throughout several decades. Here, the pair of infection and malnutrition was associated to impaired immunity and increased mortality. In this vicious cycle, individuals debilitated by infection (including gastroenteritis with diarrhea) were less likely to provide for their families and communities. Malnourished children were predisposed to an early death from infectious diseases [62]. Although the current status of food supply, as well as the life expectancy, have improved almost worldwide and infection is no longer the first cause of death in the majority of continents, with the exception of Africa [63], just a decade ago more than a third of deaths in children under five could still be associated with inadequate nutrition [64]. 

While severe malnutrition is often associated with higher rates of infection or worsening of symptoms, this is not the case for all types of enteric viruses. This was shown in an analysis data retrieved from the Global Enteric Multicenter Study, in which children with moderate-to-severe diarrhea were compared with age-matched healthy controls and classified as having acute malnutrition or better nutritional status [65]. Here, an inverse interaction was found between the association of norovirus with diarrhea and nutritional status, meaning that norovirus had a 28% weaker association with diarrhea in malnourished children than in those with better nutritional status [65]. Moreover, fatality associated with presence of every pathogen included in the study was higher among malnourished children, but in children with norovirus this increase was less pronounced [65]. A potential reduction in the expression of surface proteins required for viral access to the target cell is worthy of investigation, as it would contribute to clarify the association of malnutrition and decreased efficacy of oral vaccines, that is still a subject of controversy [66]. 

Today, in addition to protein-energy malnutrition, micronutrient supply is a focus of research for those investigating susceptibility to enteric infection as well as potential treatments. For example, an association between vitamin D deficiency (in the form of serum 25-hydroxy vitamin D3) and rotavirus-induced diarrhea was reported for Turkish pre-school children [67]. In vitro experiments using porcine intestinal epithelial cells have shown that vitamin D is able to inhibit the replication of porcine rotavirus through the retinoic acid-inducible gene I (RIG-I) signaling pathway [68], which is known to promote antiviral responses in the intestinal epithelium, including expression of type I interferons (IFN-β) [69]. Another study showed a prevalence of zinc deficiency in a population of Nigerian children suffering from diarrhea, whose stools were positive for enteric viral pathogens in 62% of cases [70].

Malnutrition is a broad term that currently includes both undernutrition as well as excess energy intake. In this regard, relatively few studies have investigated the association between the combination of energy-rich diets/obesity/overweight and susceptibility to viral infection. It has been shown that obese and overweight children and adolescents present rotavirus infection at approximately twice the rate than their lean counterparts [71]. Moreover, in children and adolescents experiencing type-1 diabetes, the onset of this metabolic disease is accompanied by enterovirus infections in 79% of cases [72]. 

**Figure 3:**
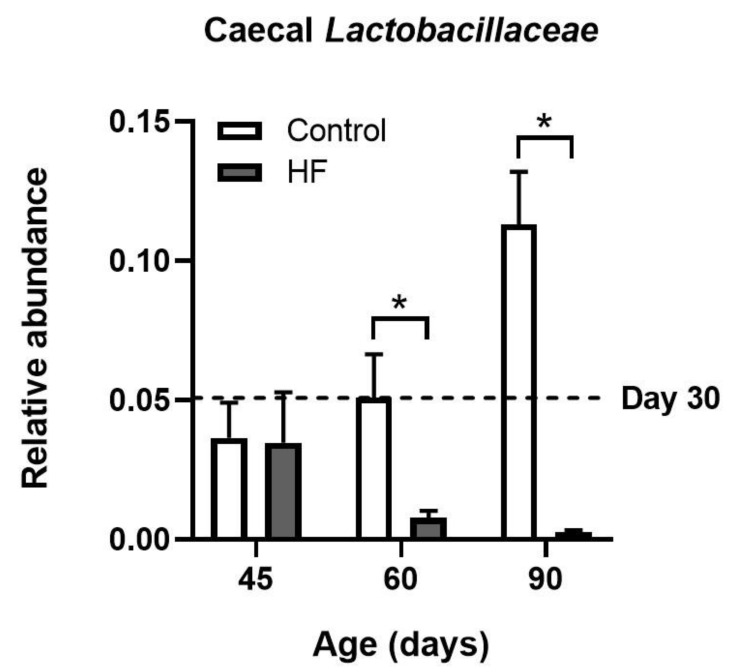
Relative abundance of *Lactobacillaceae* family in the caecal contents of rats under high fat (HF) diet. Rats received a diet containing 62% calories from fat from postnatal day 30 onwards. Age-matched controls were fed regular chow containing only 14% calories from fat. Bacterial DNA isolated from caecal contents was subjected to sequencing directed against the V3-V4 region of 16S rRNA gene, using an Illumina MiSeq platform. * *p* < 0.05 by PERMANOVA, *n* = 5. Original data from Alejandra Lopez-Aguilera and Johana Eyzaguirre-Velásquez.

Prolonged consumption of an energy-rich diet can impact gut microbiota composition, with distinct bacterial genera and species being more strongly affected (for a review, see [73]). Adding to the many effects of diet-induced dysbiosis, reductions in “good gut bacteria” could in turn, lead to opportunistic viral attack; to our knowledge, this has not been tested directly. However, we and others have found a reduction in members of the *Lactobacillaceae* family in the gut content of rodents fed a high-fat diet (see [74,75] and Figure 3). Lactic acid bacteria have been shown to protect both human and animal intestinal epithelium from virus infection through diverse mechanisms, including induction of ROS release and competition for attachment sites on the epithelium [76]. On the contrary, patients experiencing viral diarrhea have fewer *Lactobacillus* than healthy volunteers [77].

## 8. Enteric Neurons and Mucosal Barrier under Viral Attack

The intestinal tissue is innervated by two main types of neurons, both of which can undergo viral infection: a first division, characterized by their anatomical connection with distant tissues, are called intestinofugal neurons; a second subset of neurons are entirely contained within the gut tissue; these, and the glia supporting them, constitute the enteric nervous system (ENS) [78,79].

Viral effects on ENS that lead to gastrointestinal dysmotility, and therefore of concern to the myenteric plexus, have been significantly more investigated than those effects on secretion and permeability, which are functions regulated by the submucosal plexus. The investigations of Lundgreen et al. were key to demonstrate the significance of the submucosal plexus on the establishing of viral diarrhea [80]. They performed Ussing chamber experiments to measure the changes on potential difference across the gut wall, which represent electrolyte secretion and were more pronounced in tissues taken from rotavirus-infected mice, compared to healthy ones. This difference was less noticeable when tetrodotoxin or lidocaine, drugs that block nerve function, were used. The authors estimated that at least two-thirds of the mucosal secretory response to this virus could be attributed to the ENS [80]. Electrical conductance (i.e., permeability) of the gut tissue was also significantly increased by rotavirus, however in this case, pharmacological nerve blockade did not affect tissue conductance [80].

In addition, the intestine is a reservoir of neurotropic viruses, which are not necessarily classified as enteric viruses but find refuge within cells of the enteric nervous system (ENS). For example, varicella zoster virus targets human gut enteric nerves, which then become a source of latent virus able to reactivate and produce disease [81,82]. There is also evidence of duodenal submucosal and myenteric ganglia infected by herpes simplex virus type 1 [83]. Most commonly, enteric glial cells are the main targets for neurotropic viruses including HIV and adenovirus 41 [84]. The presence of such viral toxins and antigens in the ENS, in particular the submucosal plexus, could potentially disturb the mucosal barrier via over-activation of the immune response. Finally, the gut-brain axis has been implicated in SARS-CoV-2 infection. It has been proposed that the virus travels along the neurons [85]. In this sense, inhaled SARS-CoV-2 virions could access the olfactory tract, and by anterograde multisynaptic transport, potentially infect the gut through vagal fibers. The extent of interaction between the virus and the vagus/ENS remains to be unraveled.

## 9. Concluding Remarks

The interaction between enteric viruses, gut bacteria and the intestinal tissue is complex and remains an open field of scientific research. Gaining knowledge about the mechanisms implicated in virus interaction with other components of the gut ecosystem could lead to a better understanding of the pathogenesis of gastrointestinal infections and may allow for improvement of the current therapeutic strategies. The high inter-individual variability of the gut virome should be considered as a key factor for determining how the incidence of infection and the intensity of gastrointestinal symptoms can vary within a population. Even, the type/extent of interaction that the host’s cells and bacteria sustain with a potentially pathogenic virus could determine individual susceptibility to intestinal disease. In addition, studying the virus-ENS interaction and its influence on the gut-brain axis could expand our knowledge regarding (1) the impact of viral invasion on gastrointestinal mucosal functions and (2) the use of nerve pathways by pathogenic viruses from the gut in order to target the brain and vice versa.

## Data Availability

The data presented in this study are available on request from the corresponding author.
